# Food-specific sublingual immunotherapy is well tolerated and safe in healthy dogs: a blind, randomized, placebo-controlled study

**DOI:** 10.1186/s12917-017-0947-1

**Published:** 2017-01-18

**Authors:** E. Maina, M. Pelst, M. Hesta, E. Cox

**Affiliations:** 1 Laboratory of Immunology, Ghent University, Faculty of Veterinary Medicine, Merelbeke, Belgium; 2 Laboratory of Animal Nutrition, Department of Animal Nutrition, Genetics, Breeding and Ethology., Ghent University, Faculty of Veterinary Medicine, Merelbeke, Belgium

**Keywords:** SLIT, Sublingual immunotherapy, Food allergy, Dogs, Peanut

## Abstract

**Background:**

Food allergies are increasing in prevalence but no treatment strategies are currently available to cure dogs with food allergy. Over the past decade, experimental food allergen-specific sublingual immunotherapy (FA-SLIT) has emerged as a potential treatment for food allergies in human medicine. However, FA-SLIT has not been investigated in dogs. Therefore, the objective of this study was to prospectively evaluate the safety, tolerability and dispenser sterility of FA-SLIT in healthy dogs before testing it in food allergic dogs.

Eight experimental healthy beagle dogs, never orally exposed to peanut, were randomized in two groups to receive SLIT with peanut or placebo for 4 months. Subjects were monitored daily for local and systemic adverse effects. Blood samples for complete blood count and serum biochemistry, and urine for urinalysis were collected and the dogs’ body weight was recorded at day 0, 35 and 119 of the SLIT treatment. Sera for the determination of peanut-specific IgG and IgE were collected at day 0, 35, 49, 70, 91, 105 and 119. Intradermal tests were performed before (day 0) and after (day 119) the experiment. The content of each dispenser used to administer treatment or placebo was tested for sterility after usage. In order to assess the presence or absence of sensitization, dogs were challenged 6 months after the end of the study with 2000 μg of peanut extract daily for 7 to 14 days.

**Results:**

All dogs completed the study. The treatment did not provoke either local or systemic side-effects. Peanut-specific IgG significantly increased in treatment group. Even though a significant increase in peanut-specific IgE was also seen, intradermal tests were negative in all dogs before and after the experiment, and the challenge test did not trigger any adverse reactions in the treated dogs, which shows the protocol did not cause sensitization to peanut, but nevertheless primed the immune system as indicated by the humoral immune response. All dispenser solutions were sterile.

**Conclusions:**

Our results demonstrate that the used peanut-SLIT protocol is well tolerated and safe in healthy dogs. Further studies should evaluate tolerability, safety and efficacy in dogs with food allergy.

## Background

Food allergy is a relatively rare but progressive problem in both humans and dogs [1–5].

The treatment involves strict avoidance of allergen-intake and, if necessary, it is combined with symptomatic therapy. However, it is not possible to cure food allergy in dogs. Researchers have shown that allergen-specific immunotherapy may be a potentially curative treatment for food allergy in humans [6–8]. Immunotherapy entails frequent contact with the specific allergen, starting from a low dose that gradually increases. This leads to a modification of the immune response, with an increased threshold value at which clinical symptoms occur.

In humans, different approaches exist according to the route of administration: subcutaneous immunotherapy (SCIT), oral immunotherapy (OIT), sublingual immunotherapy (SLIT) and only recently epicutaneous immunotherapy (EPIT). Although SCIT has been successfully used in the treatment of patients with atopic dermatitis, its use in food allergy is dissuaded because of high risk for severe side effects such as itching, urticaria, angioedema and symptomatic bronchoconstriction [9]. To overcome these problems, OIT and SLIT have been used [10]. The former comprises the daily consumption of milligrams to grams of allergen in a food vehicle. The latter involves dispensing small amounts (micrograms to milligrams) of allergen extract under the tongue. Both these therapeutic approaches provide progressively increasing amounts of allergen, over weeks to months, until an established maintenance dose is reached. Adverse events consisting of multisystem, upper and lower respiratory tract and gastrointestinal symptoms, are reported for both routes of administration; however, in humans, the safety profile of SLIT seems superior to this of OIT [11]. Oropharyngeal itching is the most common SLIT-related side effect, which typically occurs during the build-up phase and mostly resolves without any treatment. Systemic side effects are very rare. Studies examining SLIT for specific food in humans are limited to hazelnut [12], peanut [6, 13], cow’s milk [14], peach [15] and kiwi [16]. The aims of SLIT are to achieve desensitization by increasing the threshold for clinical reactivity to the culprit food and later to induce/restore tolerance induction, which refers to the ability to ingest the food without allergic reaction after discontinuation of the therapy. Thus far, available evidence suggests that SLIT is able to induce desensitization in the majority of patients (between 52 and 100% of treated patients) with only one study reporting a lower percentage (10%) [6, 11–15]. Only one study evaluated the capacity of SLIT to induce tolerance between 10 and 50% of patients [11]. In contrast to the extensive literature describing the use of immunotherapy in humans with food allergy, no studies have been performed in dogs. Therefore, the purpose of this study was to evaluate the safety, tolerability and dispenser sterility of SLIT with peanut allergen in a prospective, randomized, blinded, controlled study in healthy dogs.

## Methods

### Study design

The study was approved by the Ethical Committee of the University of Ghent, Belgium (EC 2014/144 (experiment) EC 2014/121 (Intradermal test)).

This study was a randomized, blinded, placebo-controlled study using escalating doses of peanut extract in healthy dogs.

### Randomization and blinding procedures

Subjects were allocated to a treatment group or placebo group (four dogs per group), following simple randomization by flipping a coin: the side of the coin (heads or tails) determined the assignment of each subject. Ten equally looking dispensers were prepared by the principal investigator during the experiment. Five dispensers, named Group 1 and numbered from 1 to 5, contained different concentrations peanut extract solution, and five others (named Group 2 and also numbered from 1 to 5) contained only placebo, as described further. A second investigator, responsible for administering the solution to the dogs, and the animal care takers were blinded to the treatments.

### Animals

Eight clinically healthy laboratory raised beagle dogs were included: four intact females, one neutered female, one intact male and two spayed males. Median age was 6,25 years (±3,15 SD) (range 2–10 years) and median weight 10,5 (± 1,44 SD) (range 8,2–12,3 kg) (Table 1).

**Table 1:** Signalment and assigned group of eight beagles dogs included in the study (Day 0)

Group	No.	Sex	Age (years)	Weight (kg)
P	1	MC	9	9,8
P	2	M	10	8,2
P	3	MC	3	10,2
P	5	F	7	10,1
T	4	F	7	12,3
T	6	FS	3	12,3
T	7	F	9	11,7
T	8	F	2	9,7

Abbreviations: *F* intact female, *FS* female spayed, *M* male, *MC* male castrated, *P* placebo group, *T* treatment group

To our knowledge all subjects never received peanut in their diet. Clinical histories were evaluated and the dogs underwent an accurate clinical examination to rule out presence of allergy or other diseases, before the inclusion.

### Housing

Dogs were housed in kennels in a research facility at the Faculty of Veterinary Medicine, Ghent University. Each kennel consisted of an inner portion (90 cm × 473 cm) and an outer part (90 cm × 300 cm). All dogs had their own equipment (e.g toys and bowls) and the animal care takers were properly trained not to mix these materials among dogs.

### Peanut and placebo sublingual drops

The treatment group received lyophilized peanut extract (Greer®, Lenoir, NC, USA) fully dissolved in 50% glycerinated saline to a maximum peanut protein concentration of 20,000 μg/ml. Normal sterile glycerinated saline solution served as placebo. Both solutions (peanut extract and the placebo solutions) were poured into dark dispensers. As described previously, 5 dispensers per group (numbered from 1 to 5) were prepared. All dispensers addressed to the placebo group contained only sterile glycerinated saline solution. Dispensers for the treatment group contained increasing concentrations of peanut extract solution with number 1 being the least concentrated (Table 2). Dilutions were made with glycerinated saline under sterile conditions. Peanut extracts and dispensers containing placebo or treatment solutions were kept at 4 °C during the study period.

**Table 2:** Peanut SLIT dosing schedule for the treatment group

Week	Days	Dilutions	Dispenser n°	Pumps^a^	Protein (μg)
1					
	1	1:4000	1	1	0.25
	2	1:4000	1	5	1.25
	3	1:400	2	1	2.5
	4	1:400	2	1	2.5
	5	1:400	2	1	2.5
	6	1:400	2	1	2.5
	7	1:400	2	1	2.5
2		1:400	2	2	5
3		1:400	2	4	10
4		1:40	3	1	25
5		1:40	3	2	50
6		1:40	3	4	100
7		1:4	4	1	250
8		1:4	4	2	500
9		1:1	5	1	1000
10		1:1	5	2	2000
11		1:1	5	2	2000
12		1:1	5	2	2000
13		1:1	5	2	2000
14		1:1	5	2	2000
15		1:1	5	2	2000
16		1:1	5	2	2000
17		1:1	5	2	2000

Five dispensers (1–5) with increasing concentrations were used as well as variable number of pumps to come to increasing amounts of protein administered sublingually. The amount of protein dispensed ranged from 0,25 μg to 2000 μg. Placebo (only glycerinated solution was administered) was administered according to the same protocol

^a^Each pump dispensed 50 μl of solution

### SLIT protocol

**Fig. 1:**
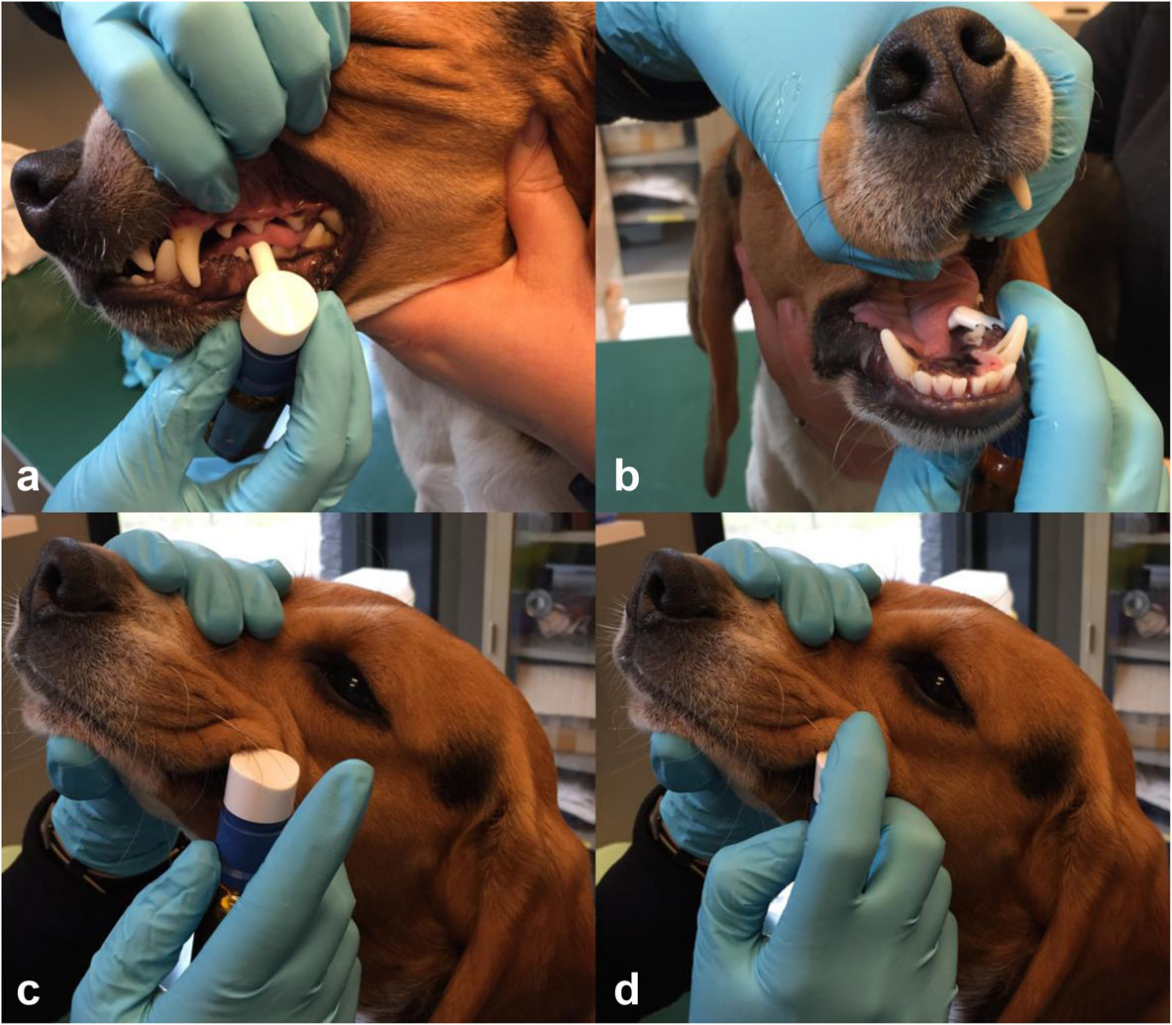
SLIT administration in a dog. Dispenser tip is hooked over the lower teeth, into the oral cavity, under the tongue **a**, **b**. A drop of solution is then dispensed by pushing the cap of the dispenser **c**, **d**

Before and during the study, dogs were fed a strict peanut-free diet. The solution was administered sublingually, by hooking a dispenser tip over the lower teeth and dispensing from 50 to 250 μl (1 push dispensed 50 μl) of solution into the oral cavity under the tongue. This was performed once a day at the same time for all dogs, at least 1 hour after the meal (Fig. 1). Dogs were not allowed to eat and drink for 30 min after peanut or placebo administration. After each administration, the oral cavity of the dogs was carefully examined to rule out accidental injuries by the dispenser. After the starting dose of 0.25 μg peanut protein, doses were increased on day 2 and again on day 3. Then weekly increases by 25 to 100% occurred until the daily maintenance dose of 2000 μg peanut protein was reached (Table 2). This maintenance dose was continued daily for 2 months. Subjects were monitored, by the primary investigator, several times during the hour following each administration. After each dose increase and the subsequent day dogs were monitored for an additional 2 h in the morning and 2 h in the evening to monitor onset of pruritus which could have been masked by the dogs’ excitement during the short daily visits. Furthermore, animal caretakers were also instructed to monitor on a daily basis for adverse effects (e.g. vomiting, diarrhoea, urticaria, angioedema and oral pruritus) and to record it.

### Tolerability assessments

#### Definition

Tolerability is referred to as absence of SLIT-related local adverse events.

#### Clinical evaluation

The muzzle, mouth and the oral cavity of the dogs were examined in detail by the principal investigator. All changes observed after the first visit (day 0) (e.g. erythema, swelling, vesicles and ulcerations, immediate or delayed oral or muzzle itching, sialorrhea, continuous chewing and vomiting) were recorded and the possible relation with the treatment was assessed by the Naranjo adverse drug reaction probability scale [17].

### Safety assessments

#### Definition

Safety refers to SLIT-related reactions that occur far from the site of administration and include both life-threatening and nonlife-threatening systemic adverse events [18].

#### Clinical adverse events and concomitant medications administered

All observed adverse events that occurred during the study period or within 14 days after the end of the experiment were recorded (e.g. diarrhoea, abdominal pain, urinary tract infection/cystitis, facial urticaria, erythema and pruritus on the axillae, groins paws and perianal area, pyoderma, otitis, epilepsy, somnolence, anorexia and anaphylaxis). Onset, duration, severity and treatments were noted. Naranjo Adverse Drug Reaction Probability Scale was used to assess the likelihood of a real adverse drug reaction [17].

#### Clinical laboratory tests

Complete blood count analysis was performed before and at the end of the experiment. Blood samples for serum chemistry, and urine for urinalysis (free catch) were collected just before the first administration at day 0, also at day 35 and again at day 119, the end of SLIT. Hepatic functions were evaluated by measurement of alanine transferase (ALT), aspartate transferase (AST) and alkaline phosphatase (ALP), and urinary functions by determining creatinine, total protein and urea concentrations. Urinalysis was performed by urine dipstick testing for pH, protein, glucose, bilirubin, specific gravity, blood, ketones, nitrite, urobilinogen and ascorbic acid.

#### Body weight change

Dogs were meticulously weighted during each visit. Any change in the body weight (BW) was recorded.

#### Intradermal test

Intradermal testing was performed at the end of SLIT, day 119, by intradermal injection of 20 μg peanut protein (0.05 ml of a 1:1000 w/v dilution of peanut protein) in the ventral lateral area of the abdomen. The wheals induced were measured after 15 min, 24 h, 48 h and 72 h. Saline solution was used as a negative control and a dilution 1:10 of histamine phosphate (0275 mg/mL) (Greer Laboratoires, Lenoir, NC, USA) was used as positive control. Peanut extract and histamine were diluted with saline solution.

### Challenge testing

In order to assess late occurring sensitization, the four treated dogs were challenged 6 months after the end of the study with sublingual administration of 2000 μg of peanut extract daily for 7 days and in one dog challenge was even prolonged for a week. All dogs were monitored for 14 days. Onset of pruritus or any other clinical signs were recorded.

### Tolerance induction assessment

#### Peanut-specific IgG and IgE enzyme-linked immunosorbent assay (ELISA)

To probe the induction of suppressive IgG antibodies rather than potentially sensitizing IgE antibodies in subjects undergoing peanut-specific SLIT, the peanut-specific IgG and IgE responses were analysed.

Sera were obtained from all dogs (active and placebo) at day 0, 35, 49, 70, 91, 105 and 119 of the SLIT treatment and then frozen at -20 °C until processed. Circulating concentrations of peanut-specific IgG and IgE were determined by ELISA. Briefly: Nunc MaxiSorp® flat-bottom 96-well plates were coated overnight at 4 °C with solutions of the peanut protein at 0.05 mg/ml in bicarbonate buffer, whereafter they were blocked at room temperature with 2% gelatine from cold fish water skin (Sigma-Aldrich®, Steinheim, Germany) in bicarbonate buffer. In subsequent steps performed at room temperature, wells were first incubated with serum samples (diluted 1/2.5 and 1/50 for IgE and IgG respectively) for 2 hours, then with polyclonal goat anti-canine heavy and light chain IgG (125 ng/ml) (Bethyl, Montgomery, USA, A40-123P) or polyclonal goat anti-canine IgE (125 ng/ml) (Novus Biologicals, Cambridge, UK, NB7346) HRP-conjugated antibodies for 1 hour and finally with a solution of ABTS (Roche Diagnostics, Mannheim, Germany). The OD was measured at 405 nm (Tecan Spectra Fluor Fluorescence and Absorbance Reader) and analyzed with the XFluor™ software. In between steps, plates were washed from three to five times, using 0,05% Tween®20 in PBS. Sera and antibodies were diluted in bicarbonate buffer with 2% gelatine from cold water fish skin.

### Sterility testing at the final container

The sterility testing was performed following the method described in the fifth Edition of the International Pharmacopoeia (http://apps.who.int/phint/pdf/b/Jb.7.3.2.pdf). Briefly, before and after first using all dispensers (placebo and treatment groups), 1 ml content was added to 10 ml of Soybean-Casein Digest sterilized Medium (Tryptone Soya Broth (TSB), Oxoid, Thermo scientific, UK) and 1 ml to 10 ml of Nutrient broth (Nutrient Broth, Difco, BD, USA). Dispenser 1 for both placebo and active treatment was also tested at day 119 to evaluate the sterility of its content overtime.

TSB was incubated at 22.5 °C and Nutrient Broth at 37 °C for 14 days. Cultures were assessed daily. In case of increased turbidity due to growth of potential contaminants such as fungal, yeast, aerobic and anaerobic bacteria, further identification occurred.

### Statistical analysis

Data were analysed with statistical software SPSS Statistics 21, (IBM, New York, United States). Haematological parameters, serum biochemistry and urinary parameters (specific gravity and pH) were compared between groups, before the experiment (day 0), at day 35 and at the end of the SLIT (day 119). The data were subjected to analysis of variance (ANOVA) in the context of general linear models at a significance level of 0.05 [19]. Summary statistics (mean and SD) for BW and percentage change from baseline were calculated at each time point. Significant differences in serum peanut-specific IgG and IgE between the two groups were calculated using a Mann-Withney U test. A p-value lower than 0.05 was considered significant.

## Results

### SLIT administration

All dogs completed the study. The administration of SLIT was easy and well accepted by the dogs. The dispensers, with their hooked nozzle, did not hurt their mucosa. During and after administration of the solutions, the dogs did not show any changes in their behaviour.

### Tolerability assessments

#### Clinical evaluation

At the time of the inclusion, all dogs were healthy and no lesions were noted on the skin and/or mucosae. All dogs but one did not show any adverse effects during or after the SLIT. Only one dog (placebo group) vomited once during the induction phase. However, according to the Naranjo scale, which estimates the probability of adverse drug reactions, this case could be classified as a ‘doubtful’ reaction to one of the components of the placebo (Naranjo score -1).

### Safety assessment

#### Clinical adverse events and concomitant medication administered

No adverse effects were recorded and therefore no additional treatment was given to the dogs during the study period.

#### Laboratory tests

Administration of peanut-specific immunotherapy had no significant effect on haematology, on indices of hepatic and renal functions nor on urinalysis between groups and over time. The values for all these parameters were within the normal laboratory reference ranges for each analyte at all time points, showing that they were not affected by the administration of either treatment or placebo (Table 3). Comparison between groups did not show any significant difference.

**Table 3:** Complete blood count, serum chemistry, and urinary values (mean ± SD) and range for eight beagles dogs included in the study at different time points

Parameters	Mean ± SD						Range
	Day 0		Day 70		Day 119		
	P	T	P	T	P	T	
*Complete blood count values*							
RBC	6,97 ± 0,72	6,39 ± 0,23	-	-	7,39 ± 0,88	6,94 ± 0,04	6,20–8,70 milj/μl
Haematocrit	47,8 ± 5,56	44,33 ± 1,99	-	-	47,45 ± 8,04	46,03 ± 1,66	43,0–59,0%
Haemoglobin	16 ± 2,21	14,88 ± 0,25	-	-	16,68 ± 2,09	15,98 ± 0,49	14,0–20,0 g/dl
MCV	68,57 ± 1,96	69,38 ± 2,23	-	-	63,95 ± 4,09	66,33 ± 2,12	63,0–77,0 fl
MCHC	33,37 ± 0,78	33,58 ± 1,55	-	-	35,4 ± 2,05	34,7 ± 0,56	30,0–36,0 g/dl
%Reticulocyte	0,43 ± 0,15	0,35 ± 0,19	-	-	0,78 ± 0,55	0,55 ± 0,33	2-0%
WBC	7556,67 ± 2091,28	7157,5 ± 1088,8	-	-	6815 ± 2775,81	8180 ± 1047	6000–16000 /μl
Neutrophil	66,23 ± 7,83	68,68 ± 1,41	-	-	62,88 ± 10,23	64,88 ± 4,84	55,0–77,0%
Lymphocyte	25,67 ± 6,13	20,98 ± 3,38	-	-	22,23 ± 2,88	19,53 ± 2,31	12,0–35,0%
Monocyte	4,2 ± 0,75	4,23 ± 0,59	-	-	6,75 ± 1,62	8,05 ± 1,23	0,0–10,0%
Eosinophil	3,17 ± 2,67	5,88 ± 3,52	-	-	7,98 ± 13,04	7,28 ± 3,36	0,0–8,0%
Basophil	0,7 ± 0,26	0,15 ± 0,1	-	-	0,18 ± 0,17	0,28 ± 0,15	0–1%
Platelet	395666,67 ± 137587,55	321500 ± 80172,73	-	-	310500 ± 63321,93	248500 ± 29949,96	164000–510000
*Biochemical values*							
Urea	2,35 ± 0,68	2,45 ± 1,05	3,2 ± 1,10	3,55 ± 1,95	2,7 ± 0,42	3,1 ± 1,70	2,5–9,6 mmol/L
Crea	28,5 ± 14,18	23,5 ± 8,54	32 ± 11,78	26 ± 9,80	24,5 ± 7,19	32 ± 28	44–159 mmol/L
ALT	24 ± 4,90	26,5 ± 5,26	33,5 ± 8,22	35 ± 8,25	10 ± 0	10 ± 0	10–100U/L
AST	11,5 ± 5	11,5 ± 5,97	13 ± 2,58	26 ± 10,95	13,5 ± 5	20 ± 7,48	0–50 U/L
ALP	108 ± 64,60	45 ± 6,63	132 ± 114,32	97 ± 43,50	122 ± 106,62	91,5 ± 69,19	23–212 U/L
*Urinary values*							
SG	1018,75 ± 4,79	1018,75 ± 2,5	1017,5 ± 2,89	1018,75 ± 2,5	1017,5 ± 5	1018,75 ± 2,5	1015–1060
Proteins (mg/dL)	Neg	neg	neg	neg	neg	neg	neg
Glucose (mg/dL)	Neg	neg	neg	neg	neg	neg	neg
pH	6,13 ± 0,48	6125 ± 0,25	6125 ± 0,25	6125 ± 0,25	6,25 ± 2,89	6 ± 0	5,5–7,0
Blood	Neg	neg	neg	neg	neg	neg	neg
Ketones	Neg	neg	neg	neg	neg	neg	neg
Nitrite	Neg	neg	neg	neg	neg	neg	neg
Urobilinogen	Neg	neg	neg	neg	neg	neg	neg
Ascorbic acid	Neg	neg	neg	neg	neg	neg	neg

*Abbreviations*: *ALT* alanine aminotransferase, *ALP* alkaline phosphatase, *AST* Aspartate transaminase, *Crea* creatinine, *MCHC* mean corpuscular hemoglobin concentration, *MCV* Mean corpuscular volume, *neg* negative, *P* placebo group, *RBC* red blood cells, *SG* specific gravity, *T* treatment group, *WBC* white blood cells

#### Body weight changes

When compared with the baseline values (placebo group: mean = 9,58 kg (± 0,93 SD); treatment group: = 11,5 kg (± 1,23 SD)), body weights remained relatively constant during the study. The mean BW at the end of the study was 9,58 kg (± 0,93 SD) in the placebo group and 11,6 kg (± 1,11 SD) in the treatment group.

#### Intradermal challenge test

Intradermal testing of dogs with peanut extract at the end of SLIT did not provoke any positive reactions.

#### Sublingual challenge test

Sublingually challenging the treated dogs with peanut extract 6 months after the end of the immunotherapy, did not provoke any clinical signs.

### Tolerance induction assessment

#### Peanut-specific IgG ELISA

**Fig. 2:**
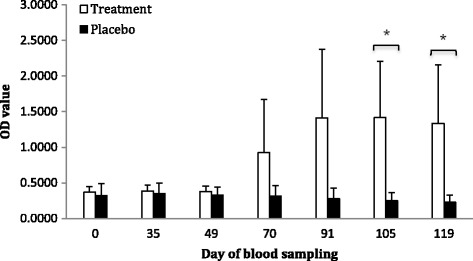
Mean increase in OD for peanut-specific IgG in the treatment (*white bars*) and placebo groups (*black bars*) ± SD. SLIT ended at day 119. Asterisk shows that at day 105 and 119, the increase in the treatment group was significant in comparison with the placebo group (*p* = 0.0267 and 0.0369, respectively)

At day 105 and 119, the treatment group showed a significant increase in peanut-specific serum IgG in comparison to the placebo group (*p* = 0.0267 and 0.0369, respectively). Although not significant at all time points, starting from day 70, the treatment group shows consistent higher peanut-specific IgG concentrations in comparison to the placebo group (Fig. 2).

#### Peanut-specific IgE ELISA

**Fig. 3:**
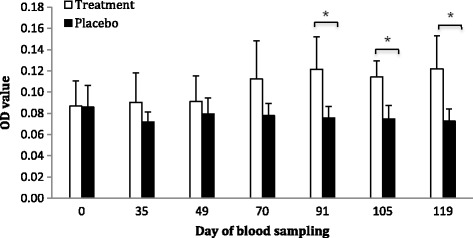
Mean increase in OD for peanut-specific IgE in the treatment (*white bars*) and placebo groups (*black bars*) ± SD. Asterisk shows that at day 91, 105 and 119, the increase in the treatment group was significant in comparison with the placebo group (*p* = 0.0298, 0.00735 and 0.0245, respectively)

At day 91, 105 and 119, statistically significant differences in peanut-specific IgE were seen when the treatment group was compared to the placebo group (*p* = 0.0298, 0.00735 and 0.0245, respectively) (Fig. 3).

### Sterility testing at the final container

**Fig. 4:**
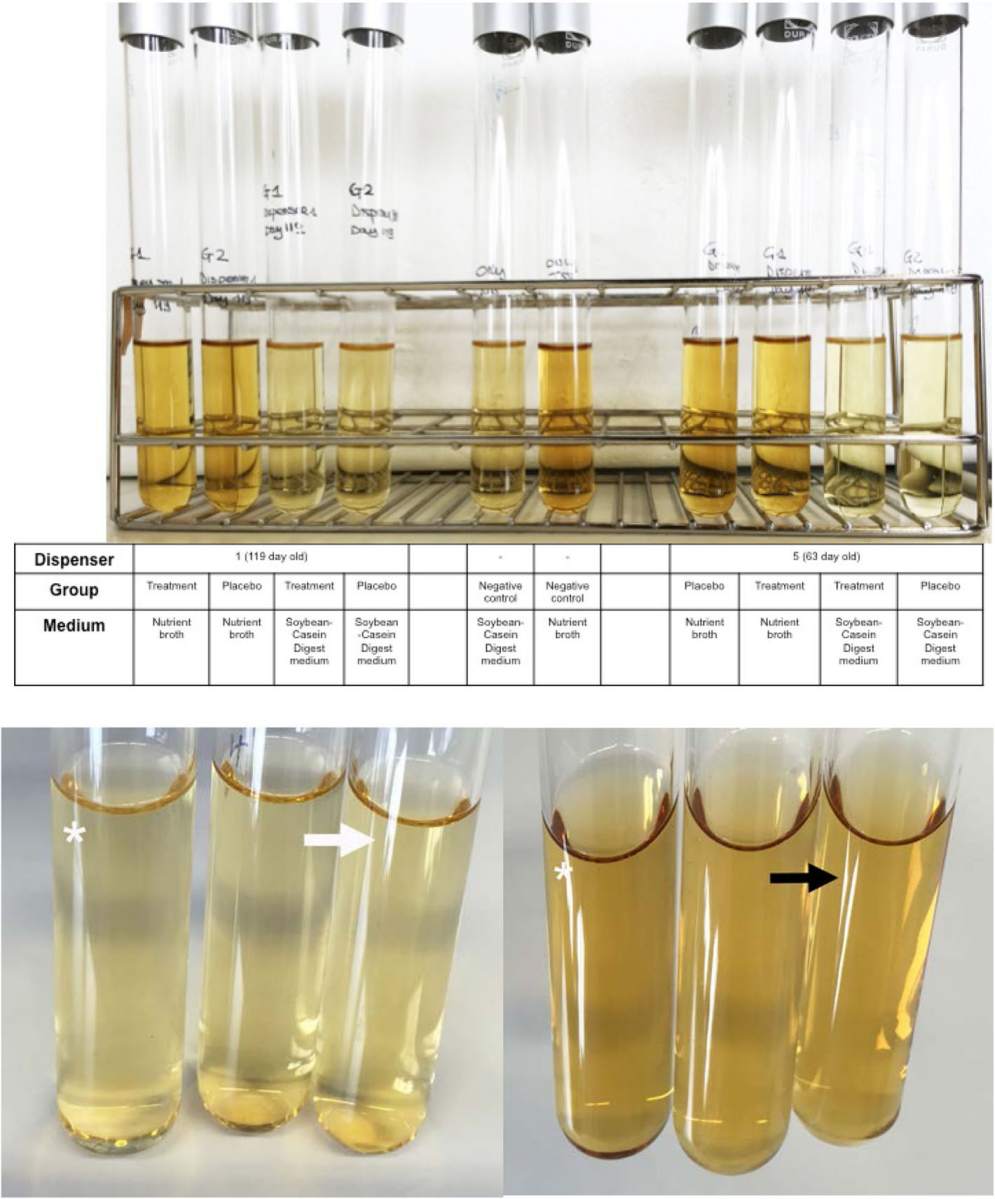
Sterility testing. Above: The content of all tubes was clear and no turbidity was seen after 14 days of incubation. Below: Magnifications of the tubes inoculated with content of dispenser 1 showing no turbidity after incubation for 14 days as described in material and methods (from *left* to *right*: Tryptone Soya Broth and peanut (*), Tryptone Soya Broth and placebo and only Tryptone Soya Broth (*white arrow*); Nutrient broth and peanut (*), Nutrient broth and placebo, only Nutrient broth (*black arrow*))

None of the culture media developed turbidity after incubation with 1 ml dispenser solutions, confirming the sterility of the tested solutions (Fig. 4).

## Discussion

This is the first prospective, randomized, blinded, placebo-controlled trial of peanut-SLIT in dogs.

This study successfully demonstrated the safety and tolerability of the peanut-specific sublingual immunization in healthy dogs: indeed, none of the dogs used in this study experienced either systemic or local side effects.

Other studies that have been performed in humans with different food extracts, including kiwi, hazelnut, peach, cow’s milk and peanut, have shown that SLIT has a very good safety profile [2, 8]. Most likely, testing different food extracts in dogs will lead to a similar conclusion.

Side effects are rarely reported in human patients and when present, they are mainly of local nature, such as swelling and itching of the lips, inflammation of the area under the tongue and the oropharynx, and often do not require treatment [6, 13]. Skin itch is also reported, but it was most commonly present in the placebo group [6]. Systemic reactions such as urticaria, angioedema and asthma rarely occur. The occurrence of side effects is mainly allergen and dose dependent and is mostly limited to the induction phase.

There are only few communications reporting the safety of SLIT in dogs. A first pilot study investigated the effect of SLIT in 10 mite-sensitive dogs with atopic dermatitis [20]. This study was followed by a multicenter open trial evaluating 124 dogs [21]. Although no side effects have been reported, it is worthwhile noting that many dogs included in these studies received concurrent medications in order to control symptoms or secondary infections. Unfortunately, there are no studies about food-specific sublingual immunotherapy in dogs. As the avoidance of the offending factors is more difficult in atopic dermatitis, it is clear that more efforts are done in searching new treatments for atopic dermatitis than for food allergy. In fact the prognosis for food allergy is generally good when the offending food allergen is identified and the dog is fed with a diet in which this allergen is absent. Strict avoidance of the offending allergen is necessary to avoid relapses. However, accidental reactions are common, as allergens can be hidden in various foods or contaminate commercial food [22]. Lack of family member’s compliance and or the inappropriate food access can lead to undesired relapses. The induction of tolerance against offending allergens might prevent such relapses and as such be an important therapy in food allergy. To be noted, while in humans one-third of the people that strictly avoided the offending food component for 1–2 years could tolerate it after such a time span, in dogs natural desensitization rarely occurs [23, 24]. Furthermore, even when the diet is strict, dogs, as human, can become allergic to another food component present in their diet after 2–3 years [25]. In humans, the sensitization to other food, also called “allergic cosensitization” or “collateral priming” has been shown to be prevented through a T regulatory-cell-dependent mechanism induced by an early allergen specific immunotherapy [26–36]. Unfortunately, this has not been assessed in dogs yet.

The goal of this study was to evaluate the safety and the tolerability of food-specific sublingual immunization in healthy dogs. Almost none of the dogs, in both the placebo and treatment group, manifested any systemic or local adverse effect. Only one dog in the placebo group vomited once; however, there was no correlation between placebo administration and clinical signs. Especially noteworthy is the extreme easiness of using pump-type hooked-dispenser bottles which allowed a fast and safe administration. In fact, we carefully examined the oral cavity and no lesions were found. In addition, absence of changes in dog’s behaviour during the study and in particular at the time of each administration, suggests that the daily administration did not affect their well-being. This is an important point which must not be overlooked because, indirectly, it may increase the compliance to treatment and consequently, also the efficacy of the SLIT.

This study was limited to healthy dogs for two reasons: first, since the main aim of this study is to evaluate the safety of the protocol, it was necessary to use healthy dogs in order to rule out irritation by the allergen used rather than a real allergic reaction. Secondly, we wanted to understand if this protocol could induce sensitization against peanut allergen, this assessment would not have been possible in dogs which were already allergic.

Peanut, *Arachis hypogaea*, was chosen because none of the included dogs had previously eaten this protein and because commercial diets for dogs normally do not contain peanut as ingredient. We must emphasize that even in case of induced allergy, the avoidance of this protein would have been easier compared with other proteins. The most striking aspect of this protein-choice is that peanut causes the most severe and typically permanent hypersensitivity reactions to foods in humans, and, therefore, it has been largely studied in human literature [37]. Currently, 17 proteins, namely, Ara h 1 to Ara h 17, have been identified as peanut allergens (WHO/IUIS Allergen Nomenclature Sub-Committee, 2015-07-07) [38]. These have been further classified as major or minor allergens based on their ability to elicit an IgE response in >90% of allergic patients [39]. Ara h1, Ara h2, Ara h6 are known as major allergens and they retain their IgE reactivity after heating and enzymatic digestion, probably due to the stable and homotrimeric structure, which protects the catalytic sites within the protein [40–44].

Since the stability and potency of allergen extracts and consequently the efficacy of the immunotherapy may be affected by contamination, solutions and dispenser preparations were made under sterile conditions [29–33]. Moreover, glycerin, which is a stabilizer and also preservative, was added to allergen extract solutions to prevent loss of allergens by sticking to the glass vials and to inhibit microbial growth.

No microorganisms could be cultured from the dispensers’ content even 119 days after preparation and when used for oral administration. Interestingly, the use of only glycerin and no other preservatives such as phenol, which is commonly used in vaccine preparations, is sufficient to maintain the solution sterile. It should be borne in mind that phenol, which is a good preservative, could denaturate allergens even when stabilised in 50% glycerine [45–48].

It could be questioned that a four months sublingual contact might not be enough to sensitize dogs. It is not possible to estimate how long this study should need to have lasted to really exclude induction of allergy, as dogs may develop food allergy spontaneously between the age of 4 months to 14 years [49]. However, high concentrations of food-specific IgE were already detected in 77,8% and in 100% of the experimentally induced food allergic beagles included in the study of Puigdemont et al. 2006, respectively at day 57 and at day 85 of the sensitization protocol, showing that sensitization can occur earlier. In addition, food-specific intradermal testing was also positive in all sensitized dogs at day 85 [50]. Even though the concentration of peanut-specific IgE was increased significantly in our experiment, the intradermal test at the end of the experiment and provocative diet challenge were negative.

To note, a rise in IgE has also been demonstrated in human studies during the initial months of immunotherapy and it does not lead to an increase in adverse reactions if a simultaneous rise in allergen-specific IgG occurs [6, 51, 52]. Interestingly, in humans, as in our study, allergen-specific IgG concentrations showed a simultaneous and more extensive increase than IgE during therapy, suggesting a good tolerance induction [6, 51, 52]. Indeed, there are many articles reporting significant increase in serum concentration of food-specific IgG and IgE after allergy specific immunotherapy (ASIT). These increases have been associated with successful oral and sublingual immunotherapy, desensitization and induction of tolerance for specific food allergens. This has been extensively reported in human literature for both atopy and food allergy and in veterinary literature only for canine atopic dermatitis [6, 53–59]. Therefore, this is the first article reporting an increase in serum concentration of food-specific IgG after administration of a new protein in naïve dogs. It is acknowledged that the skin test is not a diagnostic assay for food allergy and the oral food challenge (OFC) is still considered the gold standard test. Therefore, we performed an OFC with 2000 μg of peanut extract 6 months after the end of the experiment. None of the dogs showed any signs of allergic sensitization, further confirming that our protocol did not sensitize dogs against peanut. In a study designed to determine the minimum dose of peanut protein capable of eliciting an allergic reaction in sensitized individuals, clinical signs were evident after ingestion of 2000 μg of peanut [56, 60]. Administration of peanut during the oral food challenge lasted 7 days for 3 dogs because they were already included in a new experiment which did not allow peanut administration. It has been reported that the OFC should be continued for 7-14 days [61–64] because a small percentage of allergic dogs may require more days to show clinical signs after being fed the culprit protein. It is unclear, in dogs, if these delayed reactions require the OFC to be carried out over several days or if it is enough to administer a weight-appropriate dose of protein in a single day, as it is routinely done in human medicine, followed by monitoring the patient the following 14 days, as we meticulously did in this experiment.

## Conclusions

To conclude, we demonstrated that sublingual administration of escalating doses of peanut extract in healthy dogs is a safe and well tolerated protocol. Given the premises, this food-specific SLIT protocol might be a suitable treatment to desensitize dogs with food allergy. Future research should focus on testing the same protocol in dogs with proven food allergy.

## Abbreviations

ALP: Alkaline phosphatase
ALT: Alanine transferase
ANOVA: Analysis of variance
ASIT: Allergy specific immunotherapy
AST: Aspartate transferase
BW: Body weight
CREA: Creatinine
ELISA: Enzyme-linked immunosorbent assay
EPIT: Epicutaneous immunotherapy
F: Intact female
FA-SLIT: Food allergen-specific sublingual immunotherapy
FS: Female spayed
IDT: Intradermal test
M: Male
MC: Male castrated
MCHC: Mean corpuscular hemoglobin concentration
MCV: Mean corpuscular volume
Neg: Negative
OD: Optical density
OFC: Oral food challenge
OIT: Oral immunotherapy
P: Placebo group
PBS: Phosphate-buffered saline
RBC: Red blood cells
SCIT: Subcutaneous immunotherapy
SD: Standard deviation
SG: Specific gravity
SLIT: Sublingual immunotherapy
T: Treatment group
TSB: Tryptone Soya Broth
WBC: White blood cells.
